# Acute Dislocation of the Elbow: An All-Arthroscopic Repair of the Lateral Ligament Complex

**DOI:** 10.1016/j.eats.2023.06.014

**Published:** 2023-09-25

**Authors:** Francisco Martínez Martínez, Celia Martínez García, Antonio García López, Vicente J. León-Muñoz, Fernando Santonja Medina

**Affiliations:** a Department of Orthopedic Surgery, Hospital Universitario Virgen de la Arrixaca, Murcia, Spain; b Department of Orthopedic Surgery, Hospital General Universitario Dr. Balmis, Alicante, Spain; c Department of Orthopedic Surgery, Hospital General Universitario Reina Sofía, Murcia, Spain

## Abstract

The elbow is one of the most commonly dislocated joints. While conservative management is frequently performed for simple elbow dislocations, the importance of primary surgical treatment is still undetermined. However, promising results have been reached after surgical repair. We propose an arthroscopic surgical repair of the lateral ligament complex (LCL), performed with a horizontal suture and 2 Fibertak Knotless implants (Arthrex) placed on the LCL origin, one anterior and the other posterior. Operative treatment should be performed in patients with moderate and gross elbow laxity to avoid post-traumatic sequelae and decrease revision rates. Arthroscopic techniques create fewer complications. This procedure allows one to address intra-articular elbow joint pathology with less chance of wound complications and the ability to use bone anchors if desired.

The elbow is the second most frequently dislocated joint in humans. Up to 75% of elbow dislocations are simple elbow dislocations. Simple elbow dislocations can have devastating complications, resulting in prolonged rehabilitation, surgery, and loss of function, even after appropriate treatment. Correctly diagnosing associated injuries and avoiding prolonged immobilization are essential for preventing further complications. While conservative management is commonly promoted for simple elbow dislocations, the importance of primary surgical treatment is still undetermined. With the increasing number of retrospective studies reporting promising results after surgical repair, primary surgical management of elbow dislocations has become a pertinent topic of discussion.

## Surgical Technique

**Fig 1:**
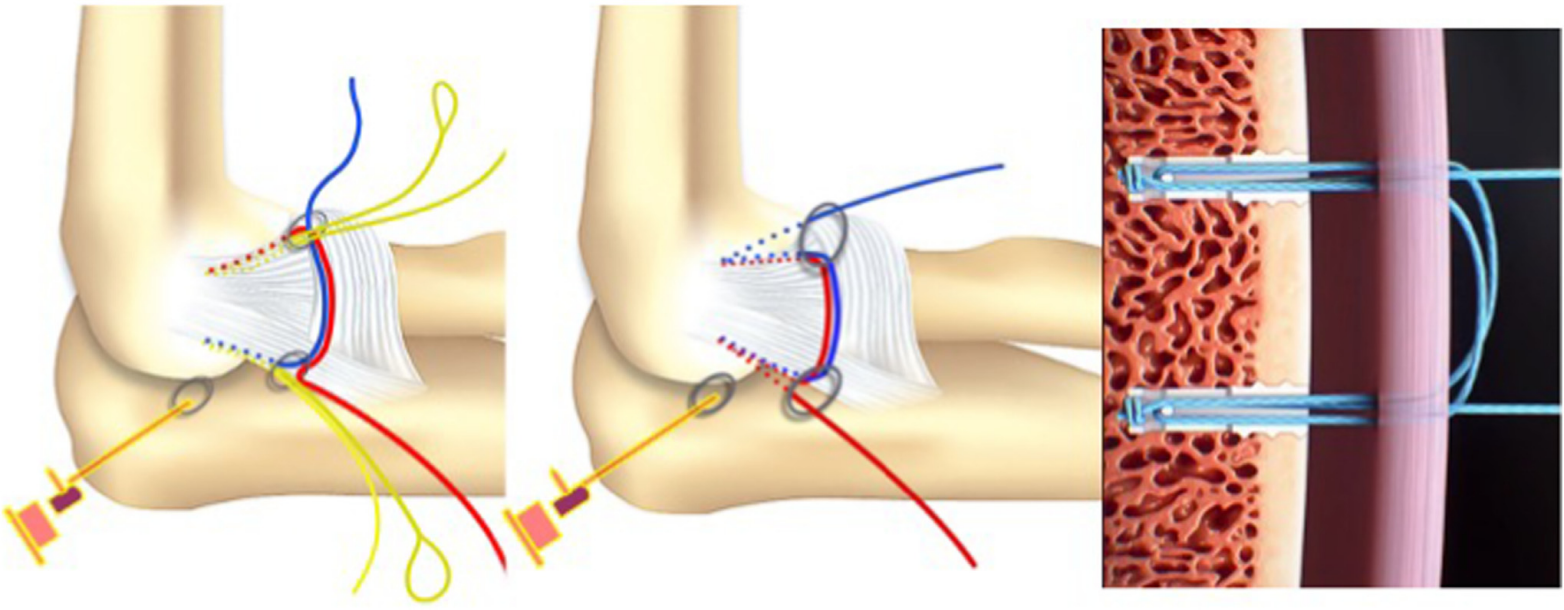
The operation is performed with the patient in a lateral decubitus position. The arm is laid on the arm rest device with the elbow placed in 90° of flexion. Two 1.8-mm Fibertak Knotless implants (Arthrex) are placed on the lateral ligament complex origin, one anterior through the anterior fascicle and the other posterior through the posterior fascicle, to perform a double horizontal suture to repair the lesion.

We propose an arthroscopic surgical repair of the lateral ligament complex (LCL) origin on the lateral epicondyle, performed with a horizontal suture with 2 Fibertak Knotless implants (Arthrex) placed on the LCL origin, one anterior and the other posterior (Video 1). The LCL has a triangular structure with a common origin in the epicondyle from which an anterior fascicle (radial collateral ligament) and a posterior fascicle (lateral ulnar collateral ligament) emerge and a communication between them that forms the annular ligament. Two 1.8-mm Fibertak Knotless implants (Arthrex) are placed on the LCL origin, one anterior through the anterior fascicle and the other posterior through the posterior (Fig 1). We proceed to exchange the limbs of both definitive sutures with the aim of making a double horizontal suture to reinsert the common origin of the LCL (Table 1).

**Table 1:** Pearls and Pitfalls

When the double horizontal suture is blocked in the implants to achieve the reinsertion of the lateral ligament complex, the indicator of sufficient tension during the tightening of the reconstructed soft tissue is the obliteration of the wide ulnohumeral joint space and the gutter.
We should avoid placing the implants very proximal to the origin in the epicondyle or close to each other in order to pick up enough tissue to be close to the footprint in the disinsertion area.
It is a reproducible and safe technique to avoid residual instabilities that can occur with conservative treatment.

**Fig 2:**
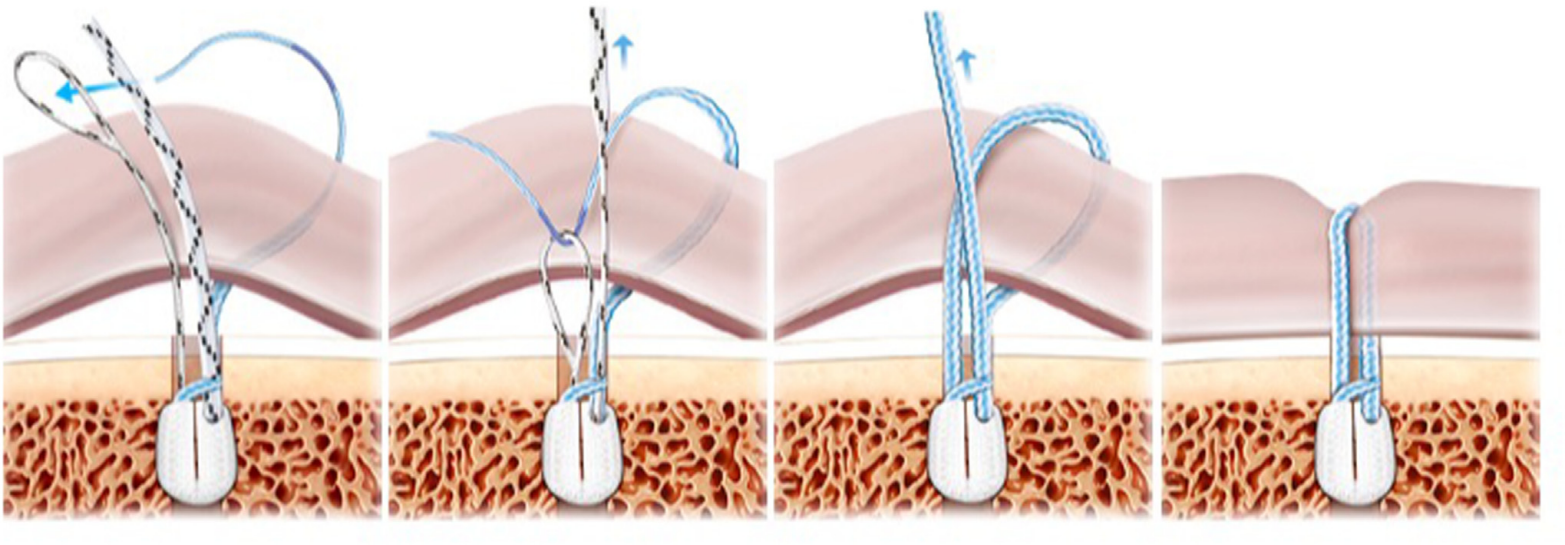
The implant consists of 3 sutures: a definitive one, which is the one that repairs the lesion, bringing the tissue closer to the implant, and two of the same color but different from the definitive one (one ends in a loop and the other not) that act as a transporter to pass the final one through the implant. With the suture passer that goes through the tissue, the loopless end of the carrier is grasped, the final suture is threaded into the loop, and, finally, the no-loop end that has passed tissue is pulled until the carrier is removed, leaving the lesion repaired with the final suture.

The implant consists of 3 sutures: a definitive one, which is the one that repairs the injury, approximating the tissue to the implant, and 2 other sutures of the same color but different from the definitive one (one ends in a loop and the other does not). They act as a transporter to pass the definitive suture through the implant. With the subcutaneous suture passer, we pick the loopless end of the transporter suture, the definitive suture is threaded into the loop, and finally we pull back from the no-loop end that has passed the tissue until the carrier is removed, leaving the lesion repaired with the final suture (Fig 2).

The operation is performed with the patient in a lateral decubitus position. The arm is laid on the arm rest device with the elbow placed in 90° of flexion. A pneumatic tourniquet is applied on the arm proximally.

**Fig 3:**
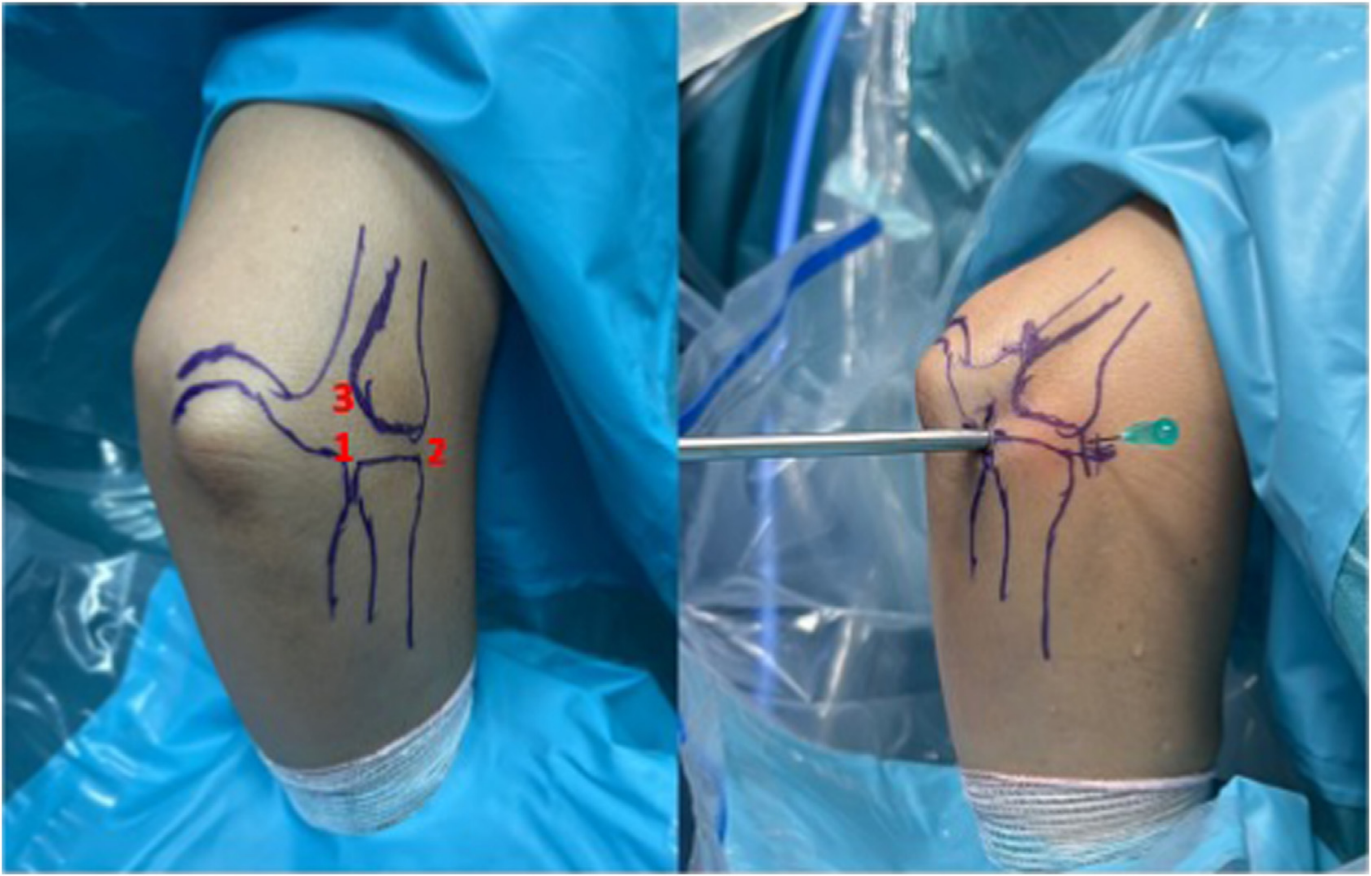
1: Soft-point portal. 2: Medium anterolateral portal 3: Accessory soft-point portal. The procedure begins with the establishment of a soft-point portal. Then the medium anterolateral portal, at the joint line level, and an accessory soft-spot portal, 2 to 3 cm proximal to the soft spot, are positioned under direct vision.

**Fig 4:**
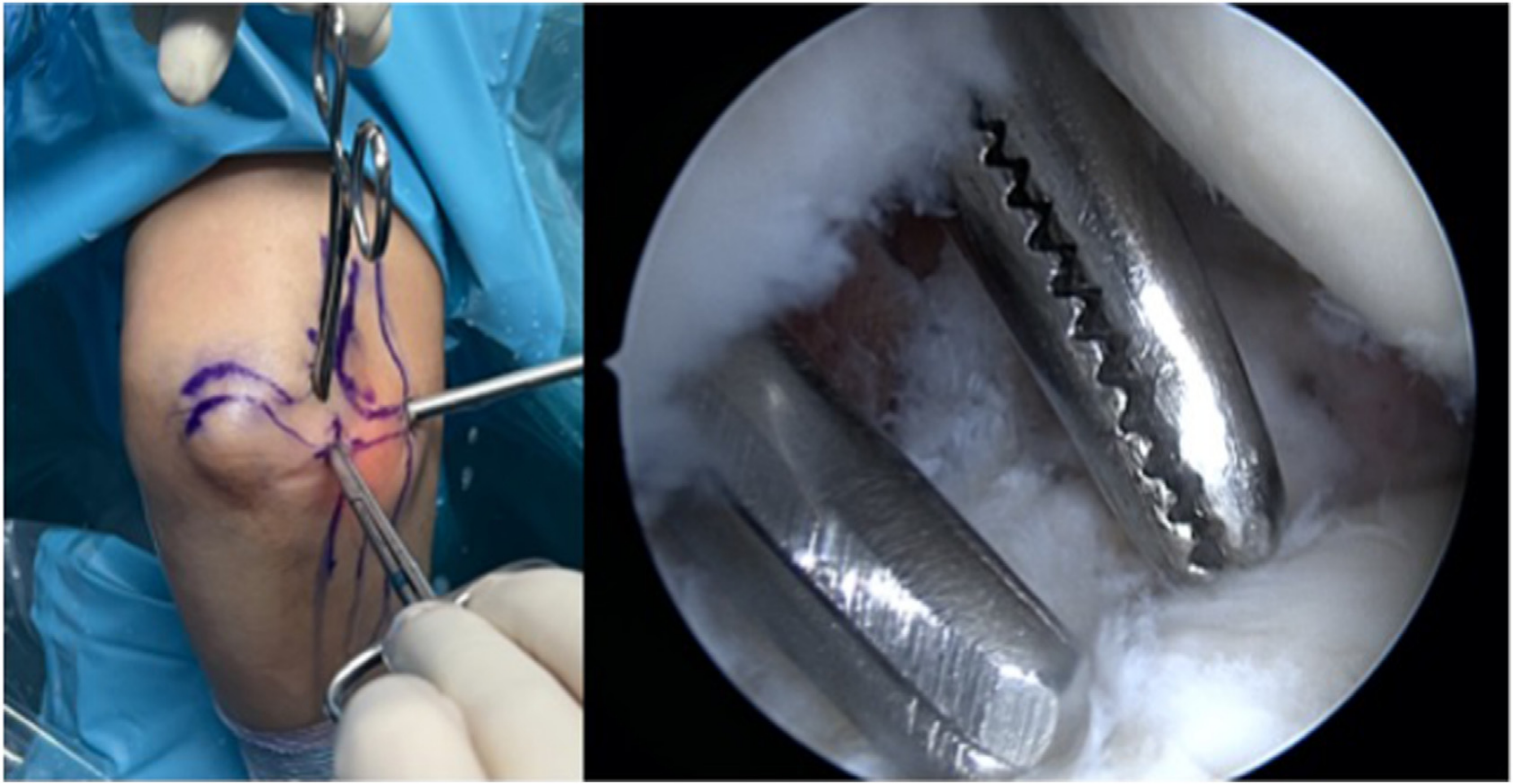
Exterior and arthroscopic image. The camera is placed in the medium anterolateral portal and the dilator is in the soft-point portal and in the accessory soft-point portal.

**Fig 5:**
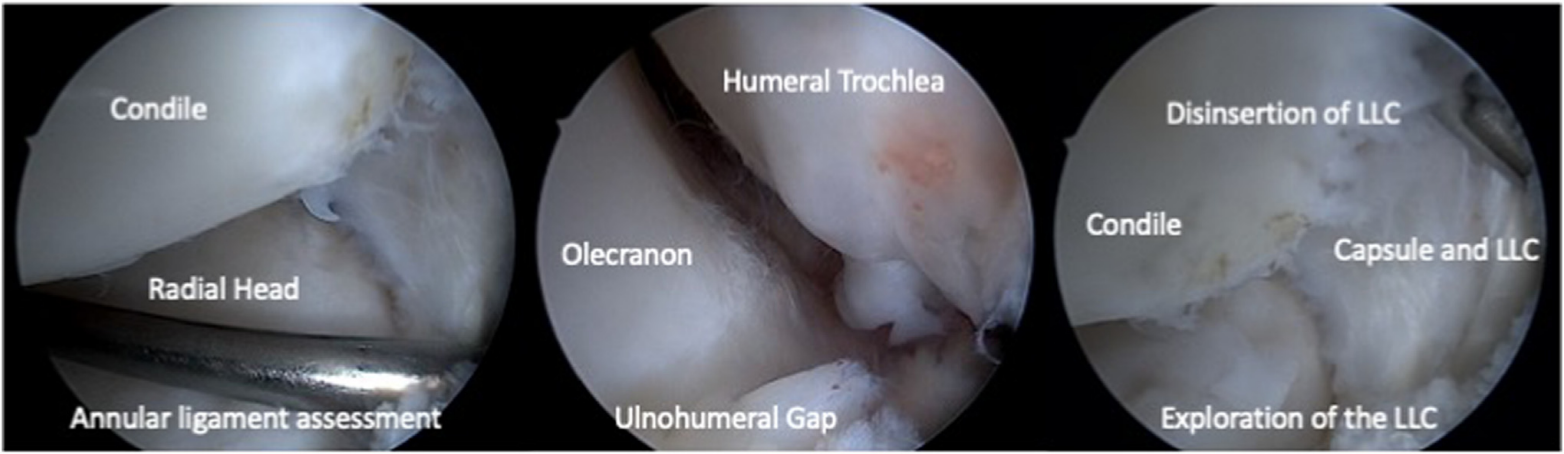
Exploration of the joint and of the disinsertion of the lateral ligament complex viewed from the accessory soft-spot portal.

**Fig 6:**
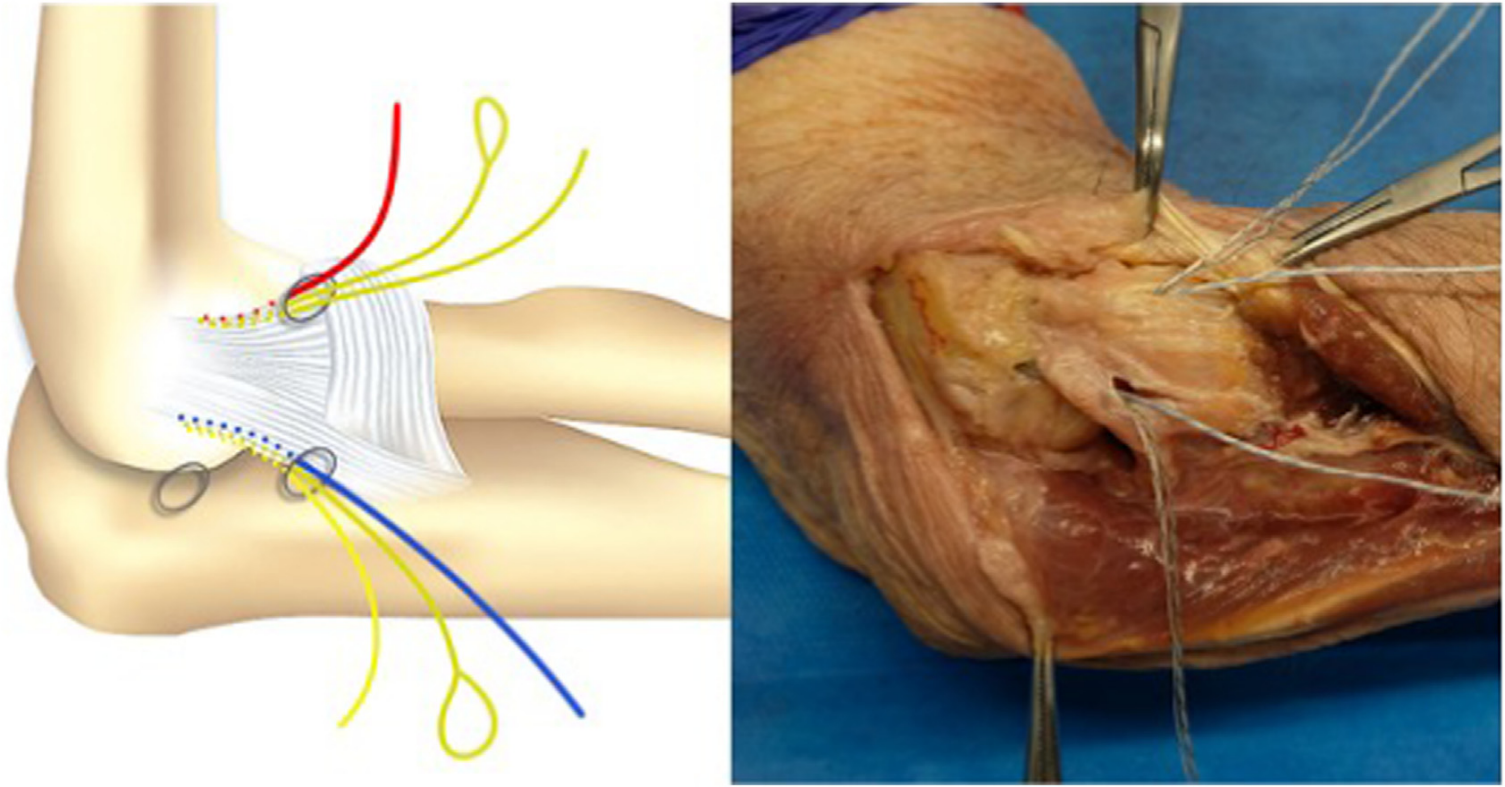
Diagram and anatomic dissection of the placement of both implants: one anterior from the anteromedial portal through the lateral fascicle of the LCL and the other posterior from the soft point portal through the ulnar fascicle of the LCL.

**Fig 7:**
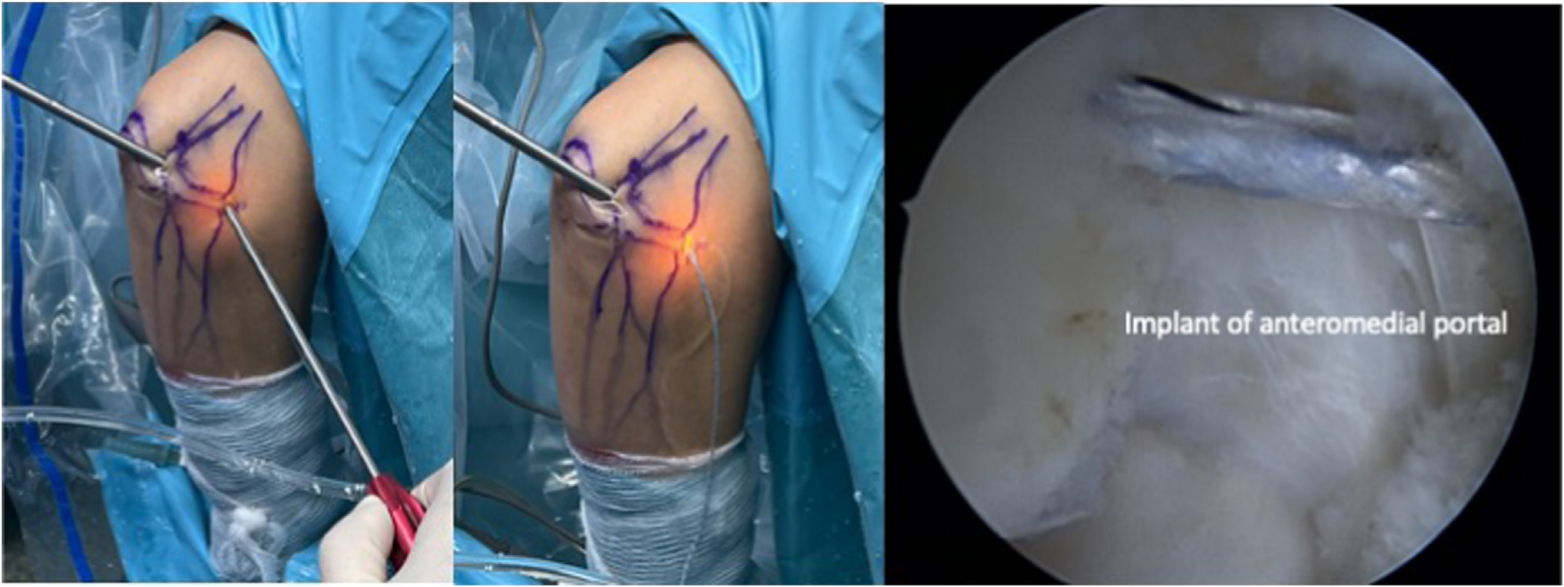
Placement of the first implant through the anteromedial portal, through the anterior fascicle, viewed from the accessory soft-spot portal.

**Fig 8:**
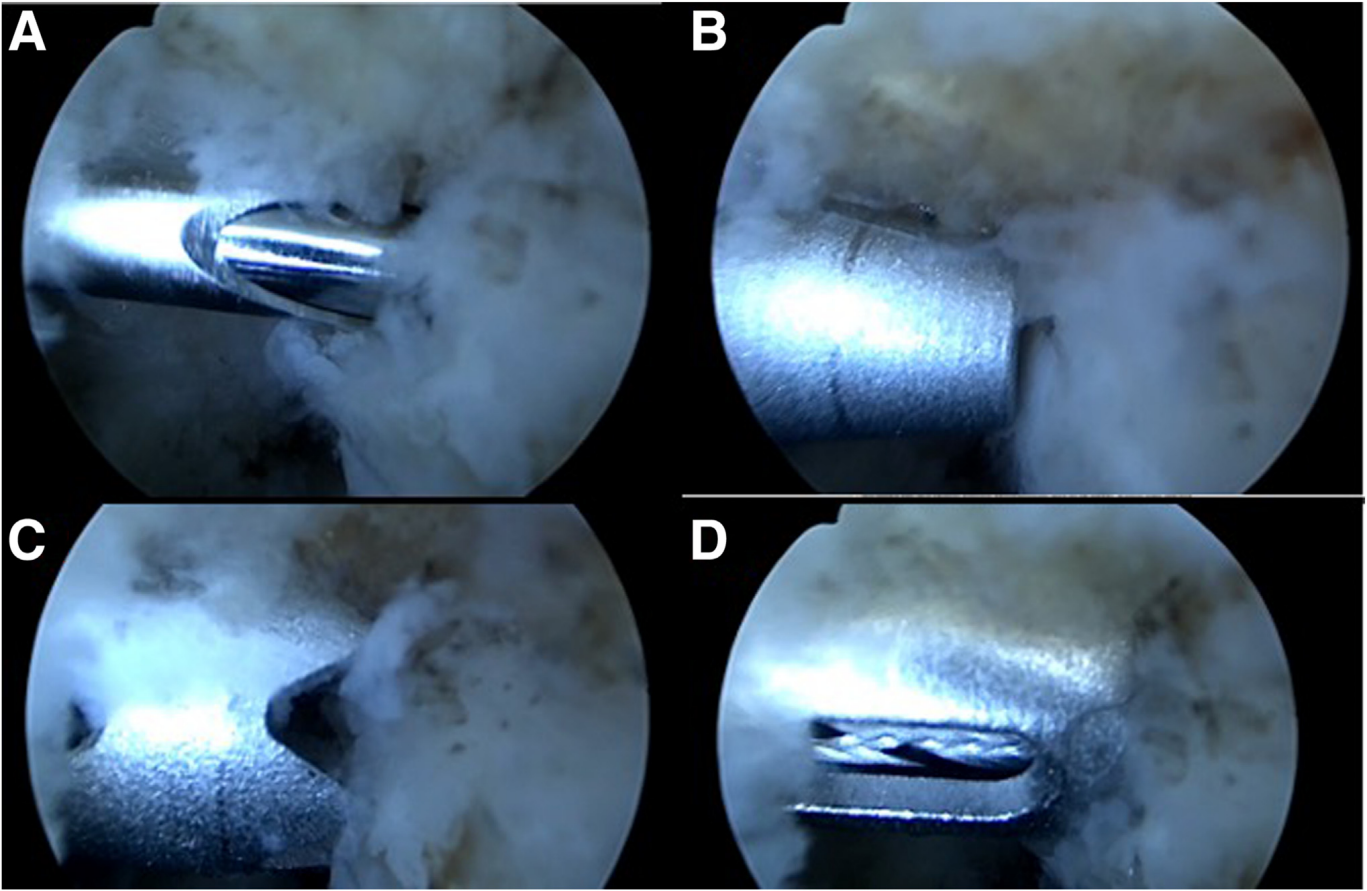
Steps for the implant placement. (a) Guide wire cannulated with nitinol. (b) A dilator is passed through the nitinol after removing the guide wire. (c) Drill guide is placed. (d) Implant placement after the brocade.

**Fig 9:**
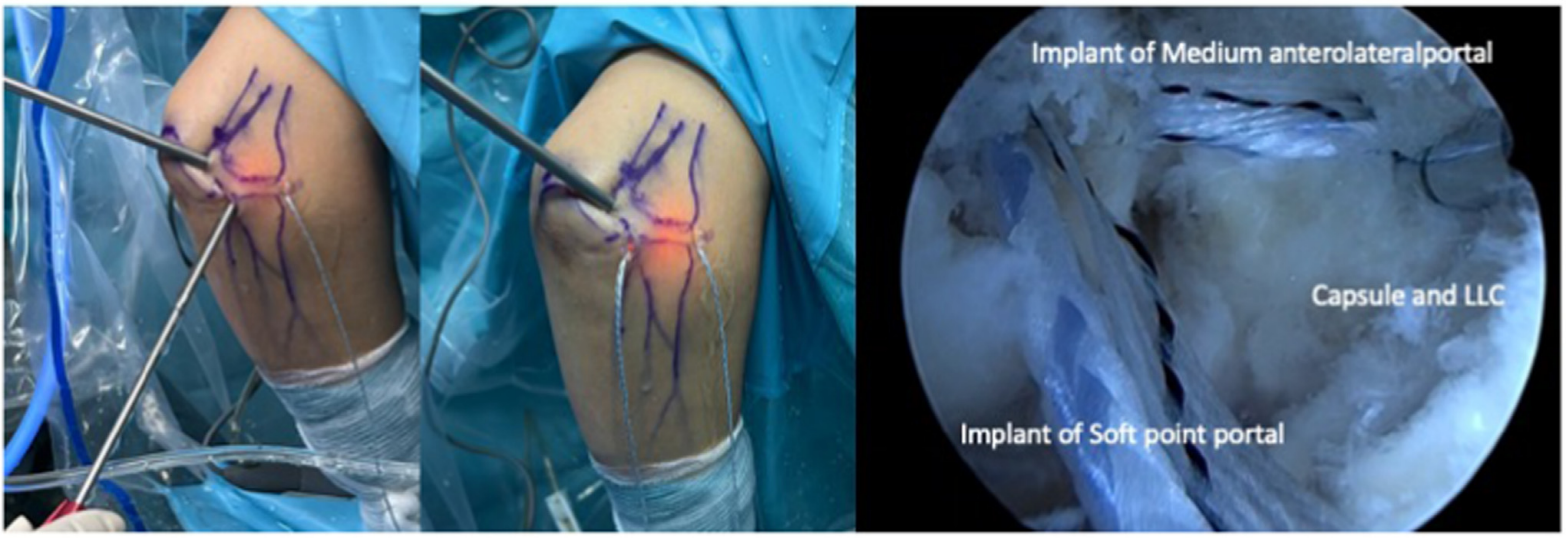
Exterior and arthroscopic image of the placement of both implants. The camera is placed in the soft-point accessory and the implants are located in the soft-point portal and medium anterolateral portal.

The procedure begins with the establishment of a soft-spot portal (Figs 3 and 4) to perform a diagnostic arthroscopy and evacuate the hematoma. The disinsertion of the LCL origin on the lateral epicondyle, the annular ligament, the proximal radioulnar joint, and possible fractures or chondral injuries can be identified (Fig 5). We can also check elbow instability, appreciating the ulnohumeral gap greater than 2 mm when valgus load and forearm supination is applied. Then the medium anterolateral portal, at the joint line level, and an accessory soft-spot portal, 2 to 3 cm proximal to the soft spot, are positioned under direct vision. The first implant, the anterior one, is then passed through the anterolateral portal (through the anterior fasciculus of the LCL) under optic control from the soft-point portal (Figs 6 and 7). Each implant starts with a guide needle. After removing the needle, we leave a nitinol wire, on which a dilator is introduced that creates a place to pass the drill guide. The drill guide is used after the brocade for passing the definitive implant, which is hammered for its introduction (Fig 8). Next, we use the accessory soft-spot portal as a viewing portal to place the second implant through the soft point (through the posterior fasciculus of the LCL) (Fig 9).

**Fig 10:**
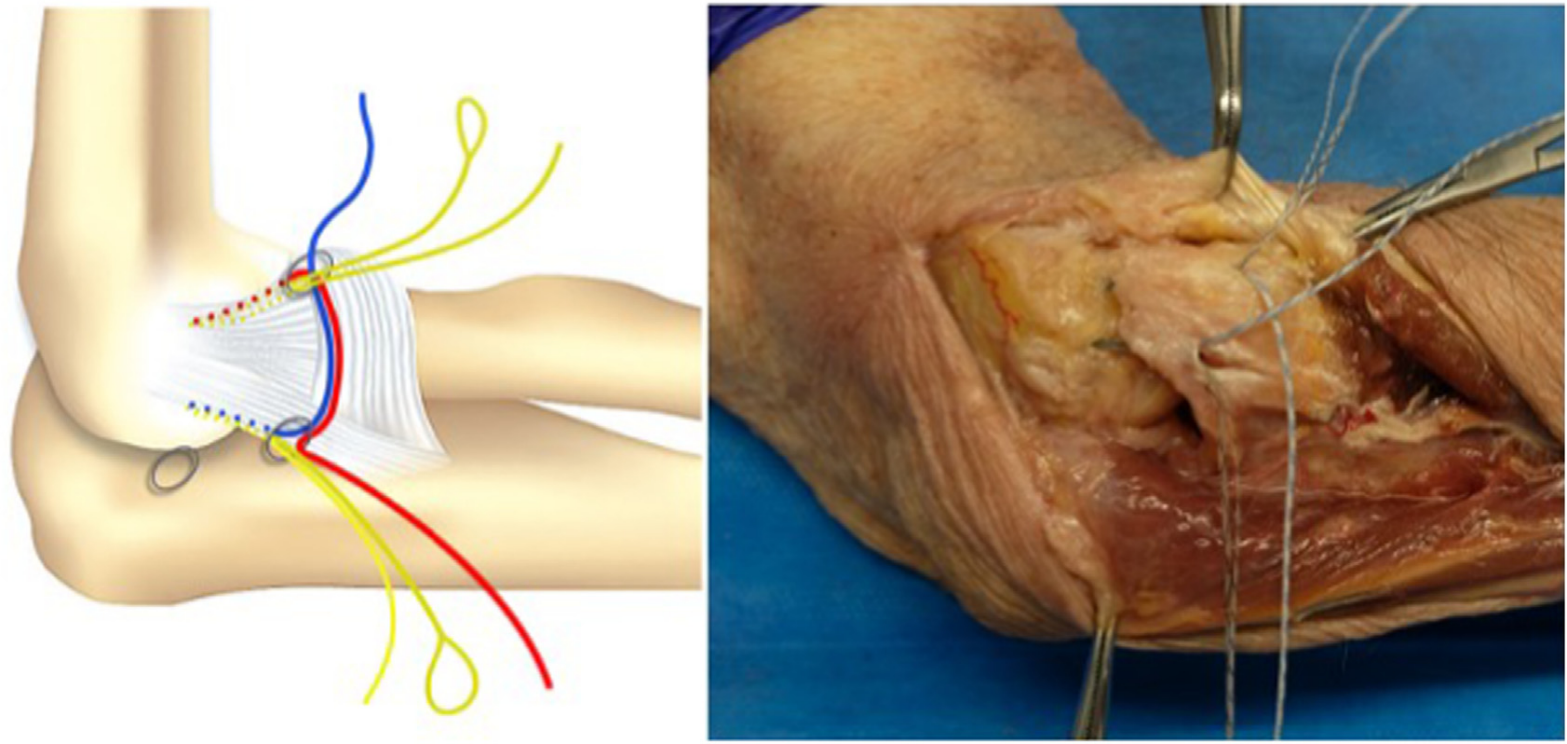
Diagram and anatomic dissection of how the final suture passes in a subcutaneous plane from the anterolateral portal to the soft-point portal and vice versa.

**Fig 11:**
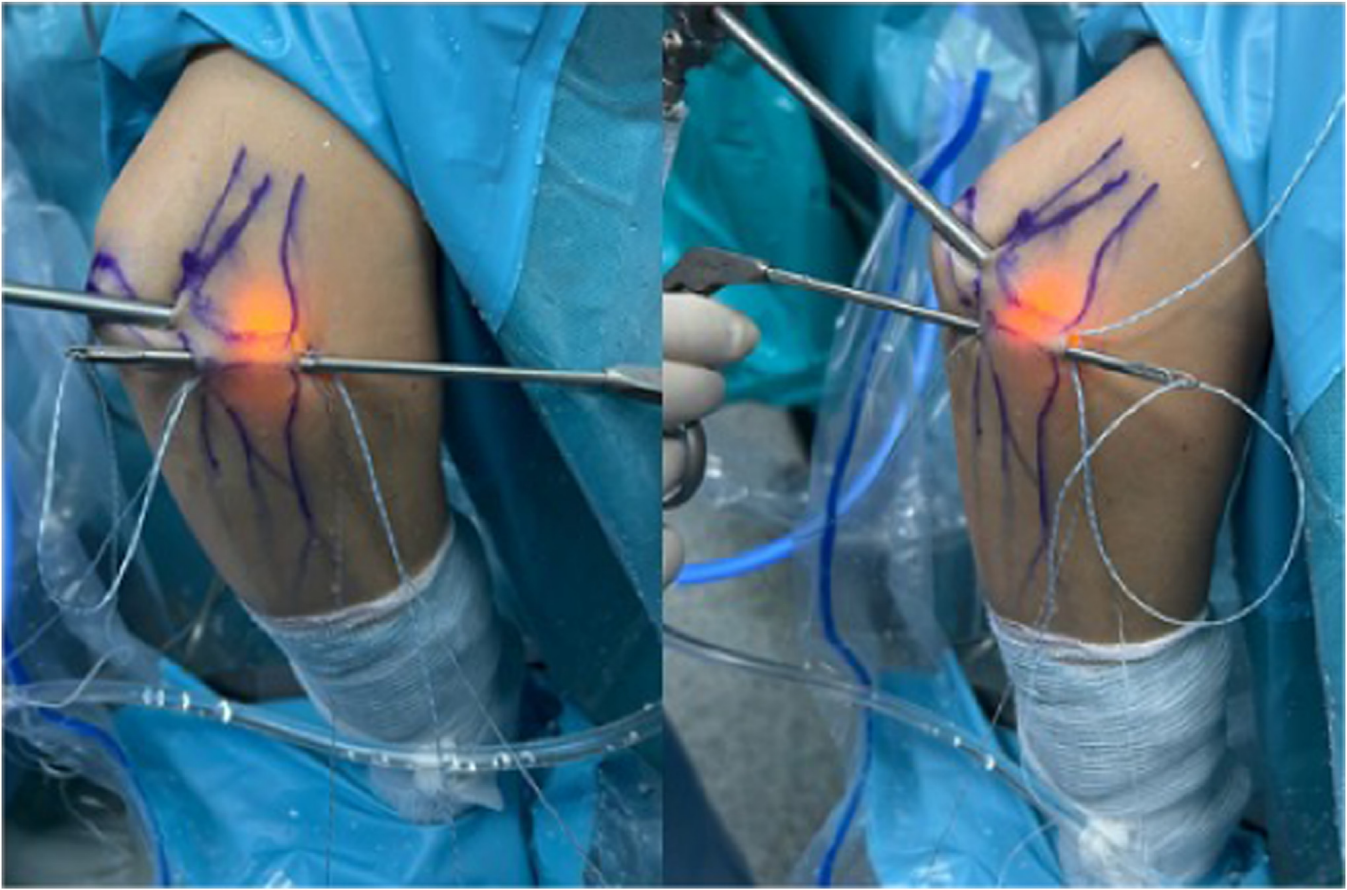
Exterior image of the final suture exchange from the anterolateral portal to the soft-point portal and vice versa.

**Fig 12:**
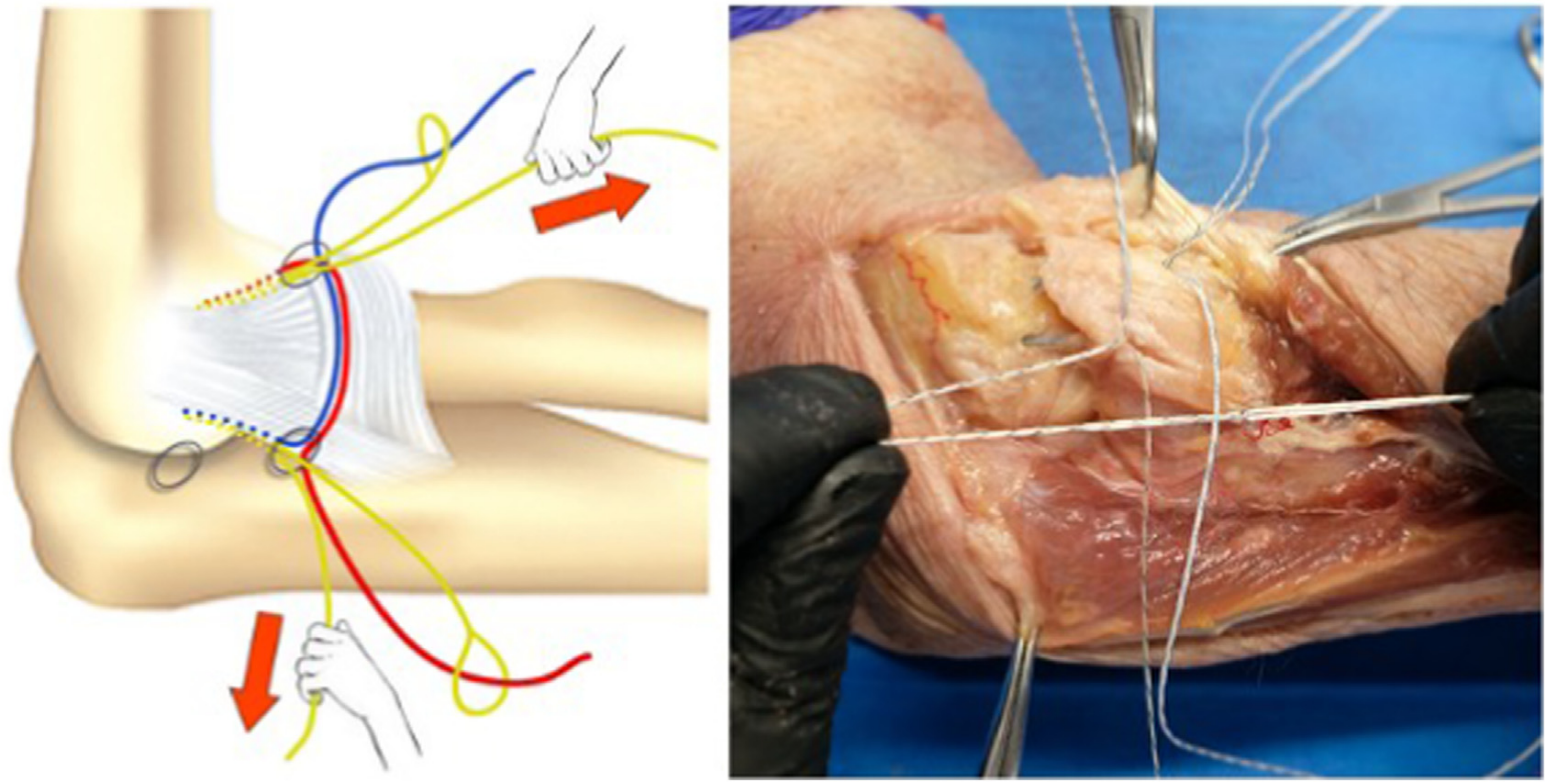
The final sutures, which have been exchanged, are introduced at the end of the buttonhole of the transporter suture and pulled at the other end until the transporter suture is removed and a double horizontal suture blocks the implants.

**Fig 13:**
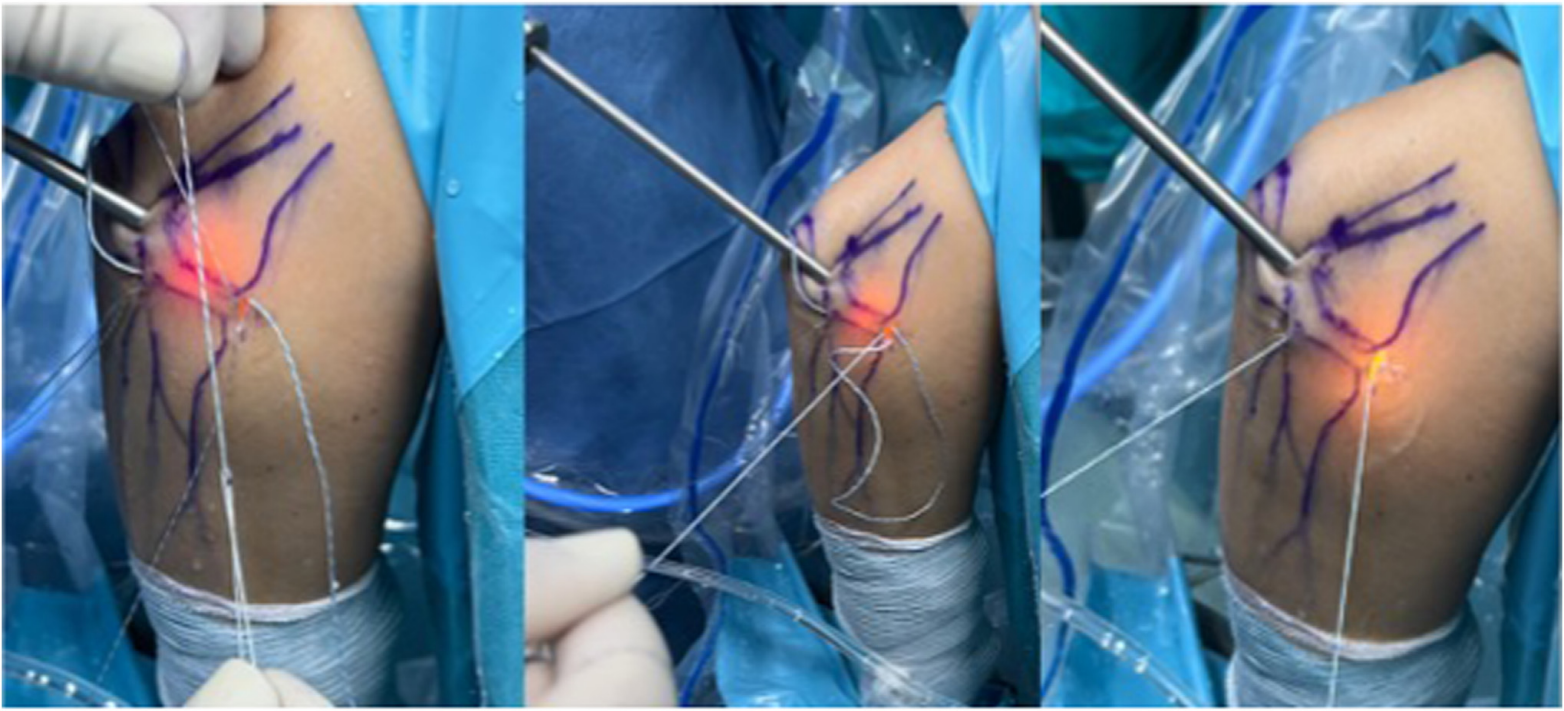
Exterior image threading the final suture of the anterior implant, which has been portal-exchanged, into the loop of the posterior implant carrier and pulling the other end of this carrier to perform a horizontal suture in the ligament as it locks onto the posterior implant. Same procedure with the other definitive suture.

**Fig 14:**
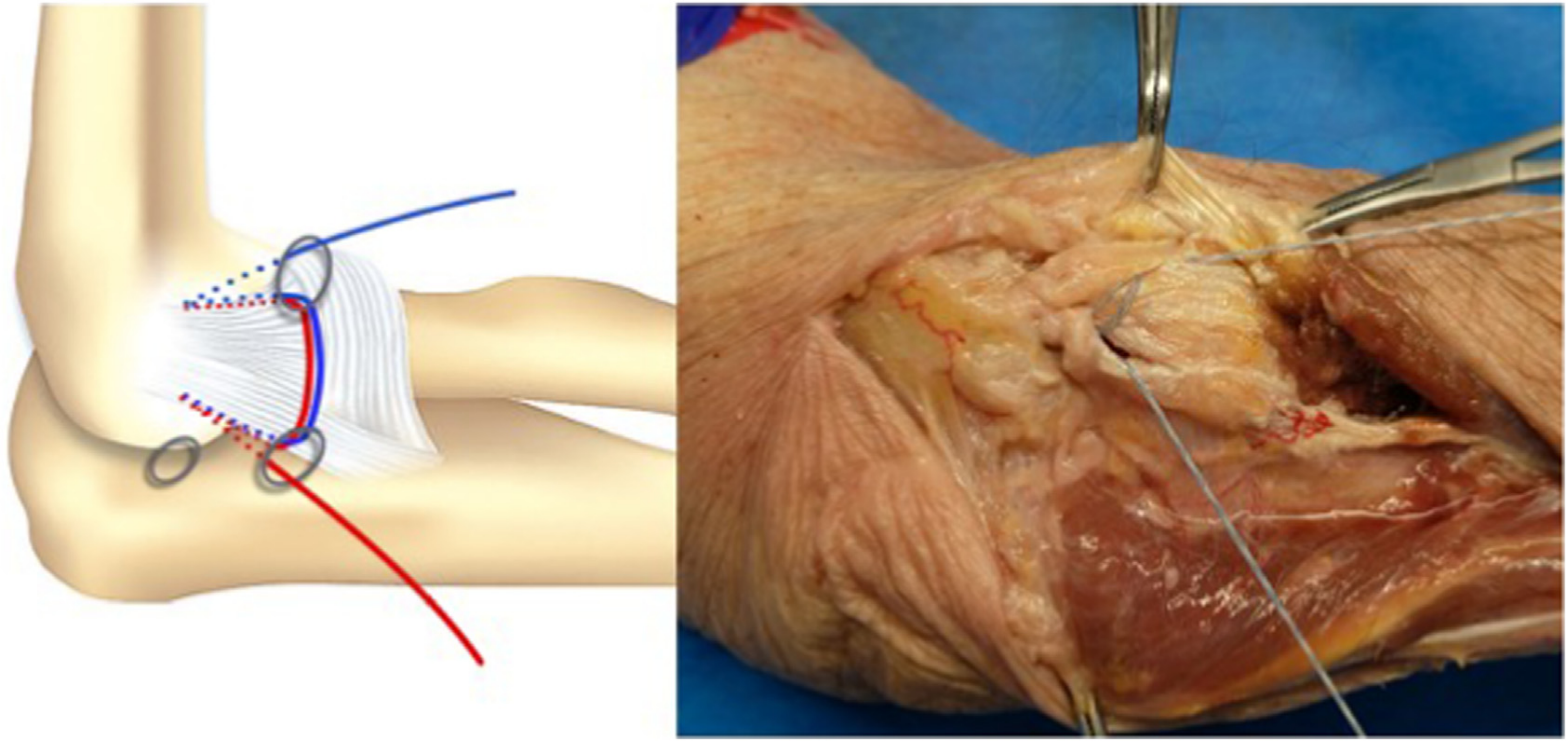
A double horizontal suture is blocked in the implants and wraps the origin of the lateral ligament complex to the lateral epicondyle to achieve the reinsertion of the lateral ligament complex.

**Fig 15:**
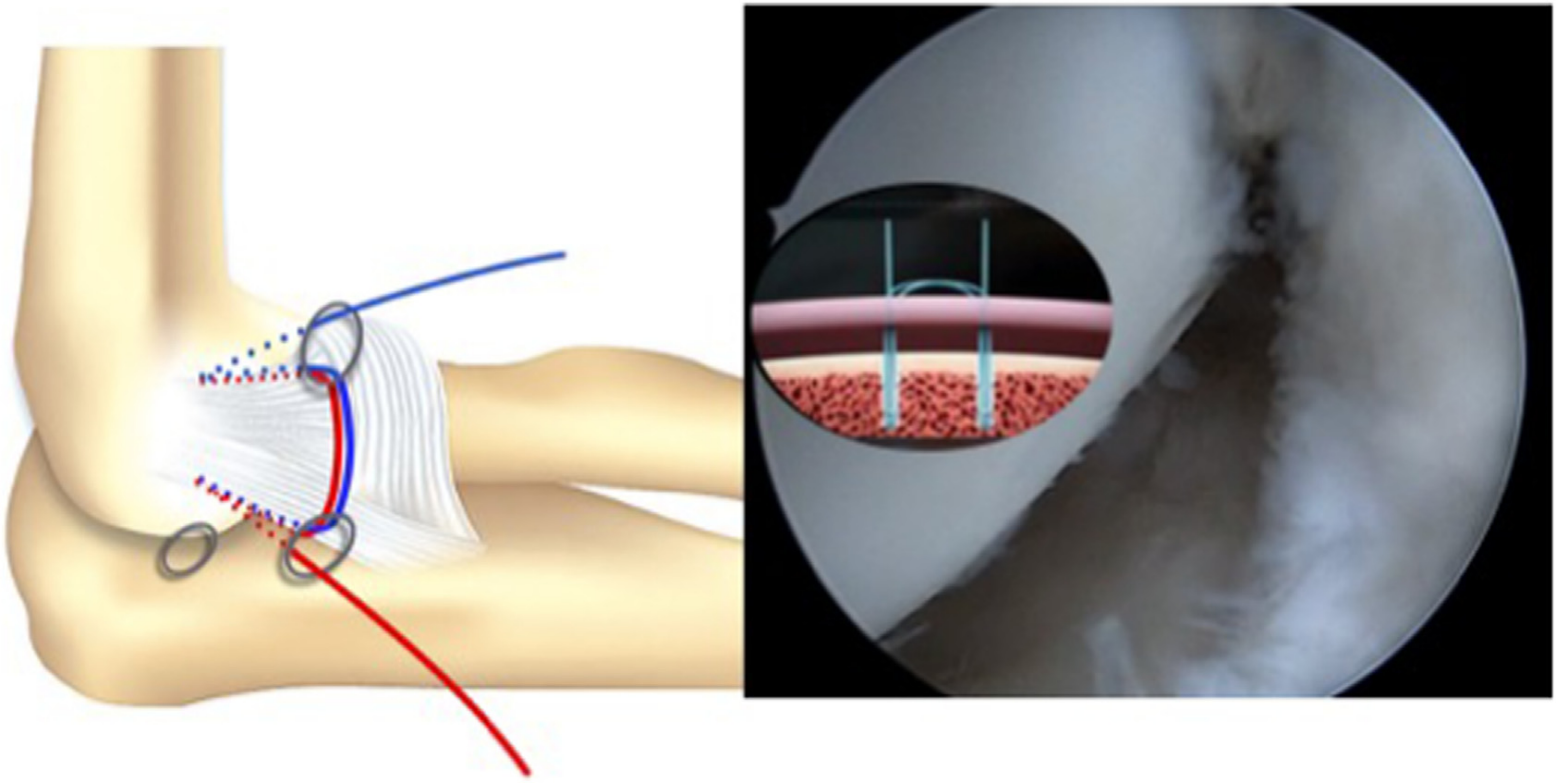
Diagram and arthroscopic image of the approximation of the tissue (capsule and lateral ligament complex) to the footprint in the epicondyle.

In order to achieve the reinsertion, a subcutaneous suture passer retrieves the definitive implant suture to the anterolateral portal through the soft-point portal and vice versa (Figs 10 and 11). The final sutures that have been exchanged are introduced at the end of the buttonhole of the transporter suture (Figs 12 and 13) and pulled at the other end until the transporter suture is removed. A double horizontal suture is blocked in the implants and wraps the origin of the LCL to the lateral epicondyle to achieve the reinsertion of the LCL (Figs 14 and 15).

### Rehabilitation

Management of elbow instability after surgery requires a fine balance between protection of all repaired structures and restoration of motion. Immediately after surgery, the elbow is immobilized using a splint in 90° of flexion and neutral forearm rotation for the first 2 weeks. Afterward, we change it for a hinge elbow brace for 4 weeks. The elbow is allowed full flexion but limited extension at 30° and 15° and full extension at 2, 4, and 6 weeks postoperatively, respectively. The isometric arm muscle strengthening is allowed immediately after removing the splint. At 6 weeks postoperatively, forearm extensor-strengthening exercises are allowed. Active and active-assisted range of motion exercises are preferred over passive exercises due to the contraction of the muscles around the elbow, which creates a joint reaction force that increases elbow stability. It is important to avoid unnecessary gravitational stresses that may stretch the LCL repair. The patient is shown how to perform active-assisted flexion and extension lying supine and with the arm overhead. Pronation and supination are practiced with the elbow in 90° of flexion keeping the arm by the side of the trunk. At 12 weeks postoperatively, pivot activities such as chair raise and pushup are allowed.

## Discussion

Most authors consider conservative management of simple elbow dislocation the standard treatment, emphasizing initial immobilization and early functional physical therapy. Conservative and surgical treatment results in good patient satisfaction and functional outcome. Surgical treatment is only mandatory when the dislocation is irreducible (<1% of cases) or when there is an inability to keep the elbow stable after reduction (10% of cases).^1^

Although patients generally report a favorable long-term functional outcome after simple dislocation of the elbow, these injuries are not entirely benign. Ten percent of conservatively treated patients complained about residual pain, restricted joint mobility,^1^ elbow stiffness, and functional instability.^1^^,^^2^ Complications of simple elbow dislocation also include contracture, heterotopic ossification, and neurovascular injuries. Mehlhoff et al.,^3^ who treated with closed reduction elbow dislocation, reported 60% residual symptoms: 15% with flexion contracture over 30°, 45% with residual pain, and 35% with valgus instability.

Surgically treated patients returned faster to their preoperatively performed activities.^1^ Thus, the choice of the treatment should be based on the stability of the elbow joint after the initial reduction,^1^ severity of the injury, and the physical demands of the patient. Operative treatment should be performed in patients with moderate and gross elbow laxity to avoid post-traumatic sequelae and decrease revision rates.^4^ The severity of the lesion on magnetic resonance imaging can also be decisive regarding the definitive choice. Also, patients with more pronounced soft tissue damage could be more likely to be treated surgically. Therefore, the higher the number of negative predictors, the greater the need for surgery as the clinical outcome will be worse, with more complications and a greater need for secondary revision surgery after conservative treatment compared to patients with slight elbow instability.^5^

Once surgery is necessary, both open and arthroscopic acute repair can be performed, with excellent patient outcomes (Table 2).^6^ Arthroscopic techniques create fewer complications than open ligament reconstruction, which result in donor site morbidity and large soft tissue dissection. This procedure allows one to address intra-articular elbow joint pathology but also earlier weightbearing, less chance of wound complications, and the ability to use bone anchors if desired.^7^

**Table 2:** Advantages and Disadvantages

Advantages	Disadvantages
1. Less donor site morbidity	1. Requires an accumulation of surgeon experience
2. Smaller soft tissue dissection	2. Technically demanding and requires precision
3. Surgeons frequently should perform an arthroscopic elbow joint examination prior to the procedure in order to confirm instability and to diagnose and treat concurrent intra-articular pathology, allowing to address intra-articular elbow joint pathology	3. Too much tension during soft tissue tightening leads to difficult range of motion exercise during the early postoperative period
4. Earlier weightbearing	4. No comparative study of biomechanics or clinical results with the previous techniques
5. Less chance of wound complications	5. Possible discrepancy between postoperative protocols
6. The possibility of using bone anchors if desired	
7. Avoidance of the graft donor site morbidities compared with tendon graft construction techniques	
8. Avoidance of violation at the osteoligamentous junction that contains proprioceptive nerve ending	

Surgeons frequently should perform an arthroscopic elbow joint examination prior to the procedure in order to confirm instability and to diagnose and treat concurrent intra-articular pathology. When a lateral open incision is created for extra-articular exploration, the benefits, as well as concept of a minimally invasive arthroscopic elbow joint procedure, are lost.^7^

However, these techniques require an accumulation of surgeon experience, they are technically demanding and require precision, and there has been no comparative study of biomechanics or clinical results with the previous techniques.^7^ Another shortcoming of this study could be the possible discrepancy between postoperative protocols.

A systematic review of repair of LCL techniques literature has been performed. In their review of the outcomes, Anakwenze et al.^4^ found that in the studies that reported the Mayo Elbow Performance Score, 91% of patients had good or excellent results. Furthermore, they noted improvements in the flexion and extension range of the elbow. Nonetheless, a complication rate of 11% was observed, with recurrent instability found in 8% of patients.

O’Brien and Savoie^8^ described 14 patients who underwent all-arthroscopic LCL repair. Patients underwent arthroscopic LCL repair using suture anchors in the humerus. Outcome scores were excellent. The patients with an acute repair returned to full activities. Athletes in the acute repair group returned to activities faster than high-demand professionals. All patients returned to their sport or profession at the same level, and none experienced residual instability.

Geyer et al.^9^ reviewed patients diagnosed with unstable elbow dislocation who were treated with primary ligament repair. Anatomic repair of the LCL was performed using suture anchor and the bone tunnel method. LCL repair alone was sufficient to obtain the stable elbow in nearly all cases. The authors concluded that the disruption of both the lateral and medial ligaments is commonly detected in unstable elbow dislocation, but surgical repair of both is not always required for all elbows. If stability can be attained by LCL repair, and early rehabilitation is permissible, nonsurgical treatment of the opposite ligament is possible.

Chanlalit et al.^10^ described a new technique but have not yet published their results in clinical practice. They performed an arthroscopic LCL reconstruction. A bone tunnel was created and prepared posterior to the lateral epicondyle for inserting a 2.8-mm PopLok Anchor (Arthrex) via the posterolateral portal. The sutures were put into the PopLok Anchor before it was applied to the tunnel. The sutures were then tightened to wrap the soft tissues to the lateral epicondyle.

We can conclude that there are no large published series on the outcomes of surgical treatment of acute elbow dislocation, and few studies have been published on results following arthroscopic reconstruction. However, studies are reporting promising results after surgical repair. We consider that the arthroscopic technique that we present is reproducible and achieves a double horizontal suture of the insertion of the LCL of the elbow that allows its healing, avoiding residual sequelae.

## Supplementary Data

ICMJE author disclosure forms

Video An all-arthroscopic repair of the lateral ligament complex.
